# Feather corticosterone levels in the southern lapwing revealed no association with the degree of urbanization

**DOI:** 10.3389/fphys.2025.1523983

**Published:** 2025-02-28

**Authors:** Verónica Quirici, Denyelle Kilgour, Elfego Cuevas, Susan Fletcher, Carlos Sarmiento, César González-Lagos, L. Michael Romero

**Affiliations:** ^1^ Centro de Investigación para la Sustentabilidad, Facultad de Ciencias de la Vida, Universidad Andrés Bello, Santiago, Chile; ^2^ Department of Biology, Tufts University, Medford, MA, United States; ^3^ Escuela de Medicina Veterinaria, Facultad de Ciencias de la Vida, Universidad Andrés Bello, Viña del Mar, Chile; ^4^ Museo Rural de Pirque, Programa Neobiodiversity, Santiago, Chile; ^5^ Departamento de Ciencias, Facultad de Artes Liberales, Universidad Adolfo Ibáñez, Santiago, Chile

**Keywords:** stress physiology, plovers, precocial, urban ecology, *Vanellus chilensis*

## Abstract

The urbanization process modifies the environment in which wildlife lives. On the one hand, it modifies the biotic and abiotic elements and introduces new stress factors like light pollution, noise pollution, and chemical pollution. These modifications to natural elements and the introduction of new ones could induce stress in organisms and lead to the release of glucocorticoids. One taxonomic group that lives in cities and is highly sensitive to changes in habitat and human population density is birds. Most of the studies about stress and urbanization have measured glucocorticoids (GCs) circulating in the blood, which offer only a “snapshot” of an animal’s current state, and it is affected by the capture procedure. An alternative is to measure GCs in samples that are not altered by the capture procedure, like feathers. In this study we compared levels of corticosterone in feather (Cort_Feather_) of the southern lapwing (*Vanellus chilensis*) in four locations in the Metropolitan Region (RM) of Santiago de Chile. To accurately measure urbanization, we employed four distinct land cover typologies to illustrate the variations in structural characteristics. A 500-m buffer zone was created around each of the four collection sites where feathers were gathered, creating an “Urbanization score”. We observed a statistically significant variation in the median Cort_Feather_ values across the four studied localities. Contrary to our expectation, the observed differences in Cort_Feather_ concentrations were identified not among the highly urbanized populations but rather between two populations characterized by lower urbanization scores. In the same line, we observed the absence of correlation between the “Urbanization score” and Cort_Feather_ levels. Our findings indicate that factors beyond those captured in the satellite images may contribute to the elevated levels of this hormone in a low urbanized wetland in the Santiago Metropolitan region of Chile. For instance, the prevalence of feral dogs in the vicinity, including within the wetland, could be a significant contributing factor.

## 1 Introduction

Urbanization, as defined by Marzluff (2001), refers to the process through which human settlements experience an increase in population density and the intensity of area use (Marzluff et al., 2001). In recent decades, the human population has grown rapidly (Pauchard et al., 2006; Grimm et al., 2008), leading to increased urbanization in both developed and developing countries (Silva et al., 2015). Urbanization is widely regarded as a severe form of habitat transformation and a significant cause of biodiversity loss (Pauchard et al., 2006; Grimm et al., 2008; Silva et al., 2015), with negative implications for many species (Pimentel et al., 1998; Biamonte et al., 2011; Jiang and Møller, 2017). Recently, ecologists have focused on studying biological diversity in highly urbanized areas (Vides-Hernández et al., 2017). Birds have been one of the most extensively studied groups in urban ecology, serving as indicators of environmental quality and playing a crucial role in the ecological studies of urbanization (Vides-Hernández et al., 2017; Leveau et al., 2017; Pirzio Biroli et al., 2020). Birds are considered an optimal model for these studies due to their visibility, ease of study, and wide distribution, as well as their high sensitivity to changes in habitat and human population density (Perepelizin and Faggi, 2009; Leveau et al., 2017; Pirzio Biroli et al., 2020; Stagoll et al., 2010).

Urbanization significantly impacts wildlife (Marzluff, 2001; Isaksson et al., 2018). It affects natural factors that influence reproduction, such as temperature, which tends to increase in cities (known as the urban heat island effect) and impacts the length of the incubation period (Partecke et al., 2006; Møller et al., 2015; Bonnet-Lebrun et al., 2020; Dominoni et al., 2020). Furthermore, urbanization results in decreased diet quality and quantity (Heiss et al., 2009; Ottoni et al., 2009; Evans et al., 2011; Isaksson, 2015; Seress et al., 2018; Peneaux et al., 2021; VanDiest et al., 2024) and increases predation pressure due to the presence of humans as a noted predator (Samia et al., 2017), For some species, urbanization reduces habitat quality and changes in nesting sites (DuRant et al., 2013). However, most studies find no differences in reproductive success, and for some species, reproductive success might even be higher in urban areas (Sepp et al., 2018; Lane et al., 2023).

Urbanization is associated with introducing novel stressors, encompassing noise, light and chemical pollution, and the emergence of diseases (Isaksson, 2015). These environmental alterations can be perceived as stressors by organisms, thereby altering their homeostatic balance (McEwen and Wingfield, 2003). Studies that have compared organisms’ physiological responses in relation to the degree of urbanization show no consistent pattern (e.g., Bonier, 2012). Four recent reviewers (Injaian et al., 2020; Iglesias-Carrasco et al., 2020; Deviche et al., 2023; Reid et al., 2024) suggest that responses to urbanization are likely to be highly species, population, and life stage-specific. Most of the studies evaluated in the revisions have measured glucocorticoids (GCs) circulating in the blood, which offer only a “snapshot” of an animal’s current state, and it is affected by the capture procedure (GCs levels are known to rise after 3 min of capture). An alternative is to measure GCs in samples that are not altered by the capture procedure, like fecal metabolites, Heterophil/Lymphocyte (H/L) ratio, and feathers or hair (Millspaugh and Washburn, 2004; Touma and Palme, 2005; Ibáñez-Álamo et al., 2020). Several studies have examined fecal metabolites (Chávez-Zichinelli et al., 2010; Buxton et al., 2018) and H/L ratio (Cavalli et al., 2018; Quirici et al., 2023) in urban and rural areas, but no consistent pattern has emerged. Some studies found no differences in fecal metabolites (Chávez-Zichinelli et al., 2010) or H/L ratio (Cavalli et al., 2018), while others reported higher values of fecal metabolites in rural areas (Buxton et al., 2018) and lower H/L ratio in a less urbanized locality (Quirici et al., 2023). Moreover, feathers and hairs are not susceptible to alterations due to capture manipulation, thus providing a more reliable indicator of glucocorticoid (GC) exposure. This advantage is attributed to their ability to accumulate GCs over the entire growth period of the feathers or hairs, presenting a longitudinal measure of GC exposure (Bortolotti et al., 2008). In birds, circulating corticosterone (CORT) (the major GC in birds- Sapolsky et al., 2000) is passively deposited in growing feathers, thus reflecting an integrated measure of CORT exposure during the period of growth, reflecting both baseline CORT levels and any increase resulting of stress events (Jenni-Eiermann et al., 2015). The quantification of CORT from feathers has already been validated by experimental studies (e. g., Fairhurst et al., 2013). Measuring corticosterone in feathers represents a “time capsule” of the corticosterone experienced by the bird during feather growth (Romero and Fairhurst, 2016) and only during feather growth (Jenni-Eiermann et al., 2015). Corticosterone in the feather is proving to be extraordinarily stable. It does not change throughout the year between molts (Wright-Lichter et al., 2021). In addition, the corticosterone is stable in the feather for over a decade when stored in the laboratory (Beattie and Romero, 2023), and can be measured from museum samples stored for decades (Kennedy et al., 2013) or even over 100 years (Kilgour et al., 2024).

Like the findings on fecal metabolites and H/L ratio, the association between urbanization and corticosterone in feathers (CORT_Feather_) does not show a clear trend: (i) higher CORT_Feather_ levels in urban localities in house sparrows (*Passer domesticus*) (Beaugeard et al., 2019) and blue tits (*Cyanistes caeruleus*) (Dominoni et al., 2021); a positive association between air pollutions (trace elements) and corticosterone in common blackbird (*Turdus merula*) (Meillère et al., 2016) (ii) lower levels in urban localities in european blackbirds (*Turdus merula*) (Ibáñez-Álamo et al., 2020) and great tits (*Parus major*) (Brodin and Watson, 2023) and (iii) no trend in Hawaiian gallinule (*Gallinula galeata sandvicensis*) (Gormally et al., 2021) and in the burrowing owl (*Athene cunicularia*) (Rebolo-Ifrán et al., 2015).

Feather corticosterone levels show no consistent trend in relation to urbanization, highlighting the need for further research. Much of the existing literature focuses on species from the Northern Hemisphere (e.g., house sparrows, great tits, blue tits, and blackbirds), and hypotheses are often generalized to other regions. However, many species in the Southern Hemisphere deviate from patterns observed in the North (Theuerkauf et al., 2022). This is particularly relevant for urban-dwelling species as urbanization progresses at varying rates, intensities, and forms across the globe (Santangelo et al., 2022). Additionally, spatial configurations differ between hemispheres: European cities are typically compact and monocentric, while South American cities often exhibit a patchy, mosaic-like structure (Suarez-Rubio and Krenn, 2018). Consequently, traditional metrics like distance to the city center may inadequately capture urbanization levels in such contexts. In this study we compared levels of CORT_Feather_ of the southern lapwing (*Vanellus chilensis*) in four locations in the Metropolitan Region (RM) of Santiago de Chile. To accurately measure urbanization, we employed four distinct land cover typologies to illustrate the variations in structural characteristics (Szulkin et al., 2020). If feathers are a good bioindicator of the stress generated by urbanization, we expect an increase in CORT_Feather_ with greater urbanization. In turn, given that in a previous study on the southern lapwing we observed that chicks exhibited higher baseline CORT (plasma) in an urban high-pollution location (Quirici et al., 2023), we asked whether these observations could be extrapolated to the feathers.

## 2 Materials and methods

### 2.1 Biology of the southern lapwings

Southern lapwings are plovers (Charadriidae). Plovers are precocial, ground-dwelling birds that exhibit variable mating patterns and flexible social structures. Their social mating systems include monogamy, polygyny, and polyandry (Lessells, 1984; Blomqvist et al., 2002). Parental care ranges from biparental care to uniparental care by either sex (Liker and Székely, 1999). Individuals defend territories either as secluded pairs (two adults) or, more rarely, in groups (>2 adults) (Liker and Székely, 1999).

Southern lapwings cover a wide geographic distribution from Central America to the southernmost tip of South America (Saracura et al., 2008) inhabit coastal areas, wetlands, fields, rivers, lake shores, lawns, and pastures, feeding on small crustaceans, mollusks, insects, and other arthropods that can be caught on the ground (Maruyama et al., 2010). Southern lapwings are 32–38 cm long and weigh approximately 250–425 g. They usually lay one clutch per breeding season during the austral winter (July, August), and they lay 2–3 (rarely 4) olive-brown eggs in bare ground scrapes. The incubation period is approximately 26 days, and fledging occurs when chicks are around 28 days old. Breeders often use the same breeding territory in consecutive seasons (Santos and Macedo, 2011). They have been described as pair-breeding and monogamous and cooperative breeding (Saracura et al., 2008), where older siblings are helpers (Cerboncini et al., 2020). The nest and young are defended noisily and aggressively against intruders through threats, vocalizations, and low flights (Santos and Macedo, 2011).

There are no detailed studies on the movement of this species, including natal or reproductive dispersal as well as migration. However, personal observations and studies in southern Brazil indicate that 90% of adults maintain breeding territories year after year (Saracura, 2003). During the non-breeding season, approximately 50% of our studied populations remain in these territories (personal observation in our studied populations). To the best of our understanding, the molting pattern of the southern lapwing remains undocumented. However, considering its classification within the plovers species, the growth period of feathers ranges from 21 to 25 days. Primary and secondary feathers molted after the reproductive seasons, so feathers collected in our study (see bellow) correspond to feather grew the previous breeding season (Pyle, 2008).

### 2.2 Study area and sample collection

**FIGURE 1:**
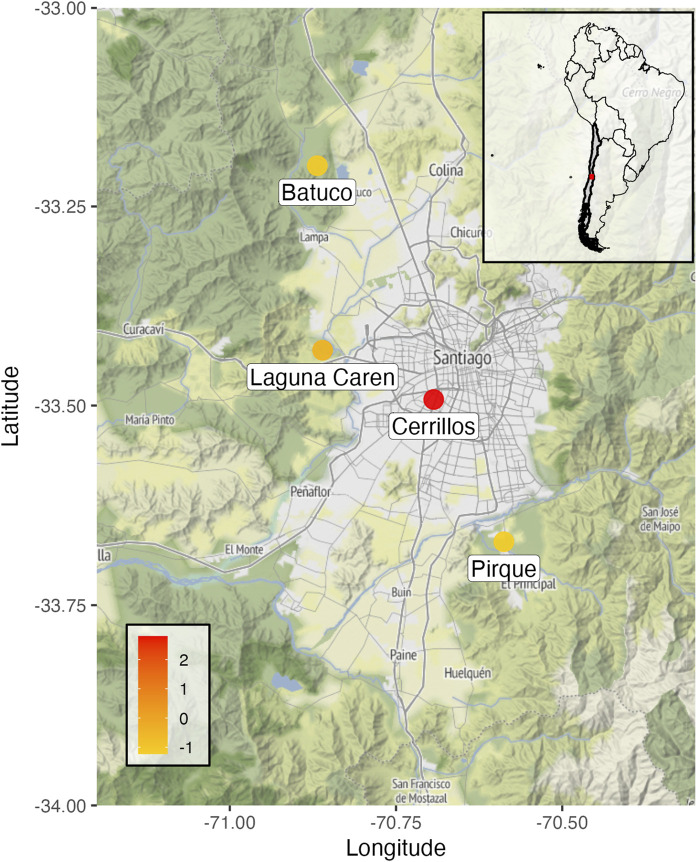
Geographical distribution of the sampling sites (Caren, Cerrillos, Pirque and Batuco) in the Metropolitan Region of Santiago de Chile.

Our study was conducted during the 2023 breeding season (December) in four localities of the Metropolitan region of Santiago de Chile (Figure 1): (i) Parque Metropolitano de Los Cerrillos (“Cerillos”) (33°29′ S; 70°42′ W), located in the south-west zone of Santiago de Chile, is a public park of 50 ha; (ii) Parque Carén (“Caren”) (33°25′ S; 70°51′ W), located in 20 km west from the downtown of Santiago, is a public park of 1, 02 ha. Although it is public, 50% of the extension is restricted to visitors (where this study was performed); (iii) Fundación Agro UC, Pirque (“Pirque”) (33°40′ S; 70° 36′ W) located 25 km south from the downtown of Santiago de Chile, is a private area of 330 ha; (iv) Wetland Batuco (“Batuco”) (33°12′ S; 70°49′ W), located 45 km north of Santiago de Chile is public wetland of 14,788 ha.

Upon arrival at the sampling sites, we conducted monitoring. Once we found a group of southern lapwing, we approached them to collect feathers. In those sites where they were grouped together, we could see the feathers molting (many of them were “stuck in the ground”) and at the same time, we observed many feces, which indicated that the southern lapwing were inhabiting that place. We collected these naturally molted feathers in the study localities (Cerrillos = 11 feathers, Pirque = 11 feathers, Caren = 21 feathers, Batuco = 23 feathers) (Table 1). To minimize the probability of selecting feathers of the same individual, we classified them based on external characteristics: recent molt (based on calamus and vane brightness and integrity), type (mostly secondaries). Individual samples were then minced and homogenized with scissors and stored into a vial. Finally, 80 mg of each feather sample were used for further corticosterone analysis.

**TABLE 1:** Habitat characteristics of the sampling sites and corresponding sample sizes. Sites are ordered from the least to the most urbanized sites (in bold: PC1 values from a principal component analysis conducted on the five habitat variables). The sites are shown in the map in Figure 1.

Sampling site (coordinates)	Habitat characteristics					Feather sample sizes
	Nighttime radiance	Built-up areas	Barren land	Vegetation	Urbanization score (PC1)	
Pirque (33°40′ S; 70° 36′ W)	16.06	0.01	0.08	0.08	**−1.23**	11
Batuco (33°12′ S; 70°49′ W)	6.02	0.01	0.22	0.10	**−1.14**	23
Caren (33°25′ S; 70°51′ W)	44.68	0.03	0.11	0.04	**−0.43**	21
Cerrillos (33°29′ S; 70°42′ W)	375.86	0.13	0.47	0.00	**2.80**	11

### 2.3 Urban characterization

To quantify urbanization at feather collection sites, we used previously classified land cover raster files. The land cover classification was divided into built-up areas, barren land, and vegetation using a single-class maximum entropy algorithm (Li and Guo, 2010) applied to 2022 Sentinel-2 images of Santiago’s Metropolitan area (Table 1). The classification utilized the annual median values of multispectral bands (B2, B3, B4, B5, B6, B7, B8, B8A, B11, B12) and 50 ground truth points from Google Earth, producing a surface-probability layer for each cover type at a 10 m resolution (Fernández and Morales, 2019; Morales and Fernández, 2020). Additionally, we incorporated nighttime radiance data from NOAA (Global Radiance Calibrated Nighttime Lights F16_20100111–20110731_rad_v4). Due to the larger resolution of this dataset (∼1 km^2^) and its diffuse landscape effects, site-level averages were calculated for all land cover types and nighttime light within 500 m buffers around each feather collection point. Calculations were performed using mland and mland_metrics from the multilandr package in R (R Core Team, 2024). The resulting statistics were combined in a principal component analysis (PCA) using the prcomp function in R. We extracted the scores for the first principal component to generate an “urbanization score” for each locality (Szulkin et al., 2020).

### 2.4 Hormone assay

Steroids were extracted from feathers and corticosterone was analyzed according to a previously established protocol (Bortolotti et al., 2008; Lattin et al., 2011). Minced and homogenized feathers were added to a 15 mL Falcon tube. Sample masses were standardized to a mass of ≥25 mg to prevent the nonlinear relationship between sample mass and measured concentration from affecting the results (Lattin et al., 2011). Then 7 mL of methanol was added to each sample tube, tubes were placed in a sonicating water bath for 30 min at room temperature, and then in a shaking water bath at 50°C overnight. The next day, feather remnants were separated from the methanol/extract solution by vacuum filtration through #4 Whatman filters (pre-soaked for at least 15 min in methanol prior to use) and Buchner funnels. Tubes, feather remnants, filters, and funnels were rinsed twice with 2.5 mL of methanol for a final volume of 12 mL methanol/extract solution per sample. Between each sample, filters were discarded and funnels were rinsed with methanol. The methanol/extract solution was dried with gaseous nitrogen while in a 50°C water bath and then extracts were reconstituted with 500 μL of assay buffer (X065 Buffer, Arbor Assays, Ann Arbor, MI, United States). Samples were then sealed with parafilm and stored at 4°C until assaying (<3 days).

The samples were then run in duplicate using a commercially available enzyme immunoassay kit (cat #K014, Arbor Assays, Ann Arbor, MI, United States) according to the manufacturer’s instructions. This kit has previously been used to analyze Cort_Feather_ (Ataallahi et al., 2020; Beattie and Romero, 2023; Branco et al., 2022; Dillon et al., 2021; Kilgour et al., 2024). Samples were run on three plates and feather pools were included on both. The inter-assay and intra-assay CV were 8.06% and 11.1% respectively.

### 2.5 Statistical analyses

We compared levels of Cort_Feather_ among localities using the Kruskal-Wallis test. We correlated “Urbanization score” with the median value of Cort_Feather_ of each location using Spearman correlation. Data analysis was performed in R software (R Core Team, 2024).

## 3 Results

**FIGURE 2:**
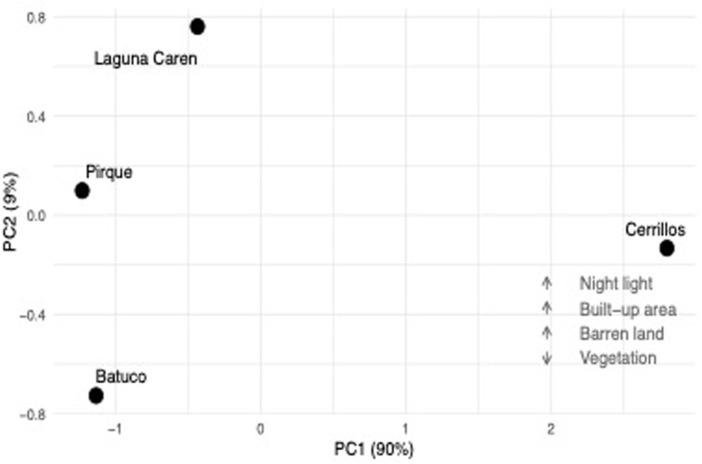
Principal Component Analysis (PCA) of the sampling localities. Urbanization characteristics are reported in Table 1.

The first principal component captures 90% of the variation in land cover statistics, indicating that Pirque is the more naturalized areas (with higher vegetation cover and lower nighttime light, barren surface and built-up areas) and Cerrillos the most urbanized area (Figure 2).

**FIGURE 3:**
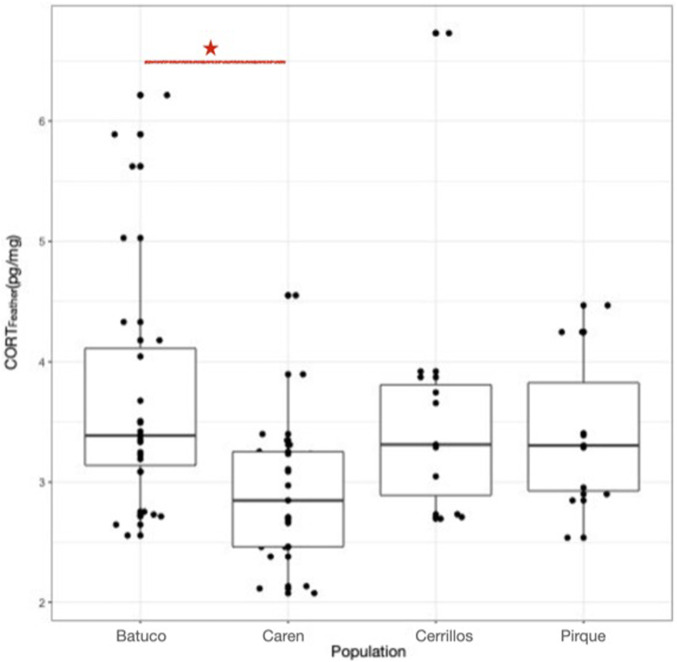
Median Cort_Feather_ (pg/mg) levels (bold horizontal line), in the four sampling sites (Batuco, Caren, Cerrillos and Pirque (Batuco presented higher levels than Caren). Top and bottom sides of each box represent 75th and 25th percentiles, respectively. Whiskers indicate maximum and minimum. The red line and the asterisk represent statistically significant differences.

We observed a statistically significant variation in the median Cort_Feather_ values across the four studied localities (H = 14.49, N = 66, *p* = 0.002). Poshoc comparations indicated that Batuco presented higher values of median Cort_Feather_ level (3.39 pg/mg) than Caren (2.97 pg/mg) (Batuco R: 41 vs. Caren R: 20.45; *p* = 0.002) (Figure 3).

We observed the absence of correlation between the “Urbanization score” and Cort_Feather_ levels (r_s_ = −0.32; *p* = 0.68).

## 4 Discussion

The findings of our research revealed variation in CORT_Feather_ concentrations across the examined populations. This observation aligns with and contributes to the existing body of literature, which has previously identified discrepancies in CORT_Feather_ levels amongst populations characterized by varying levels of urbanization. Increasing levels of urbanization have been shown to lead to higher CORT_Feather_ levels in juvenile house sparrows (*Passer domesticus*) (Beaugeard et al., 2019). In blue tits (*Cyanistes caeruleus*) nestlings, urban birds that received light at night had increased feather CORT compared with the urban dark controls, while the opposite was true for the forest birds (Dominoni et al., 2021). The contrary pattern has been observed in European blackbirds (*Turdus merula*) (Ibáñez-Álamo et al., 2020) and great tits (*Parus major*) (Brodin and Watson, 2023), rural birds presented higher CORT_Feather_ than individuals from urban habitats.

In our previous work on southern lapwings, the chicks in Cerrillos exhibited elevated levels of basal CORT in plasma compared to a rural population (Quirici et al., 2023). As a result, we expected higher CORT_Feather_ levels in Cerrillos. However, contrary to our expectations, this locality did not show higher levels of CORT_Feather_. However, the stress response (the increase in basal levels quantified 30 min after capture) was similar among the three populations (Quirici et al., 2023). So, the findings we obtained from plasma in this previous work cannot be extrapolated to feathers. This is not unexpected, as mentioned earlier, the measurement of CORT in plasma and feathers reflects different timeframes: in plasma, it captures a momentary snapshot of the present, whereas feathers encompass the baseline CORT and any increases during feather growth (Romero, 2004; Bortolotti et al., 2008; Romero and Beattie, 2022).

Conversely, the observed discrepancy between plasma (in our previous study) and feather samples (this work) from the same localities may be attributed to the differing ages at which the samples were obtained. Specifically, the plasma corticosterone levels were measured in chicks, whereas the feather samples were collected from adult individuals. So, the lack of concordance between chicks and adults at population level could be the result of the development of the H-P-A axis during ontogeny (Müller et al., 2010; Romero and Wingfield, 2015). For example, in general, in altricial species, the stress response is attenuated while the nestling is in the nest and starts to increase as the nestling approaches fledglings (Romero and Wingfield, 2015). In adult individuals, baseline corticosterone and the stress response generally decrease with age (however, see Goutte et al., 2010; Wilcoxen et al., 2011 no decrease in basal levels with chronological age), while increased stress response has been reported in senescent individuals (Goutte et al., 2010_ Eurasian kestrel *Falco tinnunculus* - Müller et al., 2010; Wilcoxen et al., 2011_ black kite *Milvus migrans* - López-Jiménez et al., 2017). Therefore, it may be plausible to hypothesize that in the region of Cerrillos, chicks exhibit a heightened responsiveness to certain stimuli, which, in their adult stage, may not elicit a similar stress response.

Furthermore, contrary to our expectations (since we previously observed that chicks exhibited higher baseline CORT in Cerrillos, Quirici et al., 2023), we noted a lack of correlation between CORT_Feather_ and the degree of urbanization as measured by our urbanization score. Interestingly, the difference we observed was not across a gradient of urbanization but rather between two populations situated within areas characterized by low urban development. Specifically, the southern lapwings population from the Batuco wetland exhibited elevated concentrations of this hormone. The inquiry thus arises as to why this area, characterized by low urban development, exhibited elevated concentrations of CORT_Feather_ compared to the other location characterized by low urbanization (Laguna Caren). Two potential scenarios were delineated to account for this phenomenon. One possibility is that feathers are not an adequate bioindicator that reflects the stress that individuals experience due to anthropogenic pressure (e.g., Gormally et al., 2021). The alternative scenario is the presence of an additional stressor within the population, one that was not detected through the analysis of satellite images. Batuco wetland is an extensive area were dogs roam freely. Their numbers are so large that they represent a public health problem (Poblete et al., 2024). They are also seen chasing nesting birds during the breeding season. This occurs constantly throughout the day and for several days (personal observation), a situation that is not observed in the rest of the localities studied in this study (personal observation). Predation pressure represents a significant ecological factor (e.g., Stracey, 2011). The presence of dogs disrupts incubation activity, causing nest abandonment in northern New Zealand dotterel (*Charadrius obscurus*) (Lord et al., 2001). Dogs’ presence also is linked to increased nest guarding in adult white-fronted plovers (*Charadrius marginatus*), but it reduces the chicks’ escape behavior, resulting in higher predation rates (Baudains and Lloyd, 2007). Therefore, dog disturbance can impose challenging conditions on wildlife, impacting potentially fitness-related behavioral responses (Baudains and Lloyd, 2007; Sheriff et al., 2009).

Given that the physiological response to escaping from predators involves an initial surge in adrenaline (milliseconds to seconds), followed by the activation of the H-P-A axis and the subsequent release of glucocorticoids from the adrenal cortex (seconds to minutes) (Sapolsky, 1987; Wingfield and Romero, 2001), it is reasonable to expect elevated levels of this hormone in this locality. Some studies have shown that the presence of dogs increases glucocorticoid levels. For example, a study by Sheriff et al. (2009) showed that in the area where the observer walked his dog, snowshoe hares (*Lepus americanus*) feces had higher levels of cortisol metabolites (the main glucocorticoid in mammals). In birds, work on the common stonechat (*Saxicola torquatus*) and song sparrow (*Melospiza melodia*) registered that baseline CORT levels in plasma were positively related to nest-predator abundance (Scheuerlein et al., 2001; Zanette et al., 2003; Clinchy et al., 2004). So, predation risk has the potential to increase the levels of this hormone. Future’s studies in our study population should registered levels of predation risk (i.e., dogs, humans) together with stress biomarkers and its consequences in parental care (aggressive territorial behavior) and fitness (reproduction and chicks’ survival) in the southern lapwing.

Given the limited sample size of this study, which constitutes a significant limitation, it is essential to gather and analyze a larger quantity of feathers to achieve more robust results. This will help ensure that the observed statistical difference between the Batuco wetland and the Caren lagoon persists. In conclusion, it would be beneficial to expand the number of populations investigated to gain a more comprehensive understanding of the impact of anthropogenic factors on the stress response of the species under study. This broader research scope could facilitate more robust conclusions regarding the ecological implications of human activities.

## Data Availability

The raw data supporting the conclusions of this article will be made available by the authors, without undue reservation.
